# The effect of a School Street intervention on children’s active travel, satisfaction with their street, and perception of road safety: a natural experimental evaluation

**DOI:** 10.1186/s12889-025-23236-8

**Published:** 2025-07-02

**Authors:** Lisa Dowling, Sophia Arthurs-Hartnett, Adriana Ortegon-Sanchez, Dan Lewer, Angela Hutton, Rebecca James, Andrew Smith, Elizabeth Bates, Sally Jones, Nicola Christie, Rosemary R. C. McEachan

**Affiliations:** 1 Bradford Institute for Health Research, Bradford Royal Infirmary, Duckworth Lane, Bradford, UK; 2 Population Health Improvement (PHI)-UK, Bradford, UK; 3 Public Health, City of Bradford Metropolitan District Council, Bradford, UK; 4 Sheffield Centre for Health and Related Research (ScHARR), The University of Sheffield, Sheffield, UK; 5 The Bartlett School of Architecture, Faculty of the Built Environment, University College London, London, WC1H 0QB UK; 6 Highways Services, City of Bradford Metropolitan District Council, Bradford, UK; 7 Sustainability Team (Place), City of Bradford Metropolitan District Council, Bradford, UK; 8 Bradford Centre for Health Data Science, Bradford Royal Infirmary, Duckworth Lane, Bradford, UK; 9 Centre for Transport Studies, Civil, Environmental and Geomatic Engineering Department, University College London, London, UK

**Keywords:** Children, Physical activity, School streets, Active travel, Built environment, Healthy streets

## Abstract

**Background:**

This study aimed to determine whether, amongst children, School Street schemes: (1) increase active travel, (2) improve satisfaction and perception of safety crossing their school street; and (3) how they are perceived more broadly by children.

**Methods:**

We recruited four intervention (School Street) and four control primary schools in Bradford, UK. Children aged 8–11 years completed a bespoke questionnaire at baseline, 4–6 weeks (T1), and one year (T2) after the intervention. Children in intervention schools were asked about their perceptions of the intervention. We used a difference-in-differences analysis to estimate the effect of the intervention on active travel, perceptions of the school road, and feelings of safety crossing the school road, with effects estimated for each intervention school separately and then pooled. Content analysis was conducted on free-text responses.

**Results:**

One intervention school withdrew and was excluded. In the remaining seven schools, 942 children at Baseline, 629 at T1, and 608 at T2 had complete data for control variables. The intervention was associated with (i) a decrease in the probability of active travel on survey day of -0.11 percentage points at T1 (95% confidence intervals -0.20, -0.02; *p* = 0.02) and -0.18 percentage points at T2 (-0.27, -0.09; *p* < 0.001); (ii) a decrease of -0.96 in the number of weekly active trips at T2 (-1.72, -0.20; *p* = 0.01); and (iii) no change in the number of frequent active travellers (≥ 3 days/week). No differences were found in children's satisfaction or perception of safety. Qualitative analysis identified three themes, School Streets: (i) increased feelings of solidarity to protect children; (ii) improved perceptions of safety by reducing vehicles outside schools; (iii) children perceived barriers to car travel.

**Conclusion:**

We saw very limited evidence that School Streets affected children’s perceptions of feeling safe, liking their school road, identifying themselves as frequent active travellers; there was some evidence for reductions in self-reported active travel. A novel finding is the sense of solidarity and community cohesion that School Streets elicits. A greater understanding of the theory of change and how the intervention works in different areas and affects different groups is required.

**Supplementary Information:**

The online version contains supplementary material available at 10.1186/s12889-025-23236-8.

## Introduction

Active travel increases physical activity, reduces the risk of obesity and developing chronic diseases, and improves mental health [1–4]. Establishing healthy lifestyle patterns like active travel in childhood that track into adulthood can reap benefits across the life course [5]. However, it is estimated that only 40% of children walk to school globally, with wide variation across countries [6]. Car-oriented changes in street design have limited children’s independent, active travel through parental concerns around traffic and safety [7], which has potentially negative consequences for children’s physical and mental wellbeing and perception of their streets. Recently, however, policies and interventions have been introduced to make streets safer and promote active travel. For example, the Healthy Streets framework, which includes 10 indicators required to improve the social, economic and environmental sustainability of streets, has been used to guide urban planning and transport policy worldwide [8, 9]. In practice, this has led to interventions involving closing streets to traffic, using technology to ‘gamify’ active travel, and changing street design [10].

Since the start of the COVID-19 pandemic, there has been a fivefold increase in the number of ‘School Street’ interventions worldwide (from approximately 200 to over 1000) and a doubling in the number of cities implementing these interventions (from approximately 60 to over 140) [11]. School Streets work by restricting access to motor traffic on the streets immediately outside schools, typically for 30–60 min at pick-up and drop-off times, with specific exemptions allowed. Restrictions are often indicated by temporary materials (e.g. cones or barriers), volunteers, or automated traffic cameras to enforce the closure. School Streets are usually installed at primary schools (ages 4–11 years) on smaller, urban, residential streets to increase physical activity through uptake of active travel (e.g. walking, cycling, scooting), reduce road danger, and improve air quality [12, 13]. Although School Streets are increasingly popular [11] and broadly, parents and guardians tend to be supportive [14], there is limited evidence of their effect on health outcomes.

The evidence to date is primarily found in the grey literature, and robust study designs are lacking. Before-and-after studies suggest that School Streets lead to reductions in traffic volume and traffic speed on the restricted street [15, 16] and that parents feel that the streets are safer [16–18]. There is some limited evidence that School Streets improve air quality around the school [16, 17]. There is a paucity of evidence regarding whether School Streets improve the uptake of active travel, which is one of the main reasons local authorities introduce School Street schemes [14]. In Birmingham, 20% of parents perceived an increase in children actively travelling to school [15]. One and two-year before-and-after studies suggest 3–9% increases in active travel [16–18]. However, the study in London had a low response rate (3%) across 36 schools (19 intervention, 17 control schools) [17], whilst the study in Oxfordshire reported a high loss to follow-up (76% of baseline) [18]; both coincided with the COVID-19 pandemic. The evaluation of schemes in Edinburgh used an ad-hoc travel tracker with unclear methodology (e.g. response rate, dates of travel) [16].

To date, studies examining the effect of School Streets on active travel have been small and have used a before-and-after methodology, which is unable to account for wider trends which may influence behaviours and attitudes (e.g., the impact of COVID-19). Moreover, these studies have sought to evaluate the impact of School Streets from the viewpoint of adults (parents, teachers, and residents). As such, it remains unclear whether School Streets achieves one of its primary aims, improving active travel uptake, considering wider trends. It is also unknown whether children, who are the primary target group of these interventions, feel safer or more satisfied with their school roads or whether children report different outcomes.

This study builds on previous evidence using a controlled study design. This study aimed to determine whether School Street interventions (1) increase active travel amongst children and (2) improve children’s satisfaction with their school road and perception of safety crossing the street to their school. A secondary aim of this study was to contextualise these findings by asking children about the changes School Streets brought about and how these changes might relate to children's health and wellbeing.

## Methodology

### Ethical approval and consent

This evaluation was conducted in accordance with the Declaration of Helsinki. Ethical approval was granted by the University College London (UCL) Research Ethics Committee (ref: 4129/008). Informed consent was obtained from the legal guardians/parents of study participants via an opt-out process. Parents and legal guardians received a participant information sheet via their school outlining the project and data collected and which instructed them to inform their child’s teacher if they did not wish their child to take part. Assent from eligible children (study participants) was obtained prior to data collection.

### Study design

This evaluation was conducted in Bradford, UK, in 2023/24, Four intervention and four control schools were recruited.

Surveys were completed at three time points:

- Baseline: May 2023-July 2023
- Time 1: October 2023; 4–6 weeks post-intervention
- Time 2: May 2024; 12 months post-intervention.

### Setting

Bradford is England's fifth largest metropolitan district, with a population of over 546,000 (Office of National Statistics, 2022). Residents of the city are predominantly of white British (57%) and Pakistani origin (25%) [19]. A third of the population lives in the most deprived decile of neighbourhoods in England [20].

### Participants

Participants included children in Years 4–6 (age 8–11 years) at Baseline and children in Years 5–6 (age 9–11 years) at Time 1 and 2.

### Intervention (School Street)

The template for intervention description and replication (TIDieR) checklist was used to describe the rationale and delivery of the School Street intervention or scheme (also referred to here as ‘School Streets’) [21]. Control schools did not receive any intervention.

#### Why

Following assessment, engagement, and successful application of the 18-month experimental traffic regulation order, Intervention School 1 and 2 launched in June 2023, and Intervention School 3 in September 2023. All three schools aimed to ease traffic congestion and the associated risk to pupil safety and targeted pupils, parents, residents, and other community members.

#### What (materials and procedures)

 The Local Authority gave each school resources to restrict traffic, such as portable signs and cones, safety equipment, high-visibility jackets, and a toolkit of resources to help the school promote the project, such as template letters. Permanent pedestrian and cycle zone 618.3 C road signs indicating “entry to, and waiting in, a pedestrian and cycle zone restricted” were installed on lampposts at the School Street entrances, advertising the times the restrictions were in operation [22].

All intervention schools began the School Street scheme by having staff place cones and portable signs on the road and stewarding the restricted zone. This ensured that exempted vehicles, such as emergency vehicles, were given access. Council Wardens, Police and Police Community Support Officers supported the implementation by sporadically visiting the three School Street sites and warning road users not to contravene experimental traffic regulation orders. The School Streets team within the Local Authority maintained contact and advised the schools throughout the intervention. During termly updates, schools provided anecdotal evidence of implementation issues to the Local Authority and research team.

#### Who provided

School staff were trained by the School Streets team (the Local Authority) on safely placing cones and signs on the road. The School Streets team was comprised of Local Authority officers with expertise in Highways, Public Health, and Air Quality. Police, Police Community Support Officers, and other council staff (e.g. ward officers, neighbourhood wardens, and parking services) provided ad hoc support to the school staff implementing the scheme.

#### How, where, when, and how much

Designated School Street roads had restrictions on motor traffic at drop-off and pick-up times for 30 min at Intervention Schools 2 and 3, and for 20 min at Intervention School 1, on weekdays during school term time. Intervention Schools 1 and 3 had two closure points, resulting in a restricted road length of 30 m and 80 m, respectively; while Intervention School 2 had four closure points, resulting in restrictions on 200 m and 225 m of the road.

#### Tailoring

In liaison with the School Streets team, schools tailored the road closure times, duration (minutes) and length (metres) before implementing the scheme.

#### Modifications

Following reports of altercations with road users, Intervention School 2 staff stopped stewarding the restricted zone entrance shortly after October 2023. Local authority staff (e.g., ward officers, neighbourhood wardens and parking enforcement officers) and Police Officers worked alongside school staff for two weeks in January 2024 in an attempt to re-launch and raise awareness of the scheme and improve compliance. After this date, Intervention School 2 staff only placed signs and cones at the restricted zone entrances when staff resources and capacity allowed but did not actively steward the area. Council staff and Police continued to deploy to the area on an ad hoc basis thereafter and continued to offer support as capacity allowed. Intervention School 3 complemented the introduction of the School Street intervention with an independent walking home policy for older pupils whose parents provided authorisation.

#### How well

Anecdotal barriers to implementation identified by the Local Authority included: (a) resistance by residents at Intervention School 2, (b) lack of clarity around vehicle exemptions, (c) perceived traffic displacement on neighbouring roads, especially at Intervention School 3, which was perceived as a crucial issue as Intervention School 3 is located next to an arterial road into the city. Anecdotal enablers included: (a) the willingness and availability of staff to continue stewarding, which was a critical facilitator at all three sites, (b) effective communication with the community, (c) ongoing support from the Local Authority and school community, and (d) engaged and enthusiastic headteachers who championed the School Street intervention and regularly communicated with their school staff, which was reported particularly at Intervention Schools 1 and 3.

### Recruitment

#### School Streets

Highway engineers at the Local Authority determined the feasibility of a School Street intervention at each primary school in Bradford District. All schools were rated as red (not feasible, for example due to presence of bus routes, heavy traffic, a doctor’s surgery), amber (some challenges to feasibility, for example due to moderate traffic flows or multiple closure points required) or green (likely feasible, for example due to generally cul-de-sac or low-trafficked routes). The current level of active travel in each school was not considered in the eligibility criteria. The scheme was advertised to selected schools that were rated green; and primary schools made expressions of interest to the Local Authority. The Local Authority invited staff from interested schools to an information session in November 2022, where they could ask members of the School Streets team at the Local Authority questions and ease concerns. During the session, the team explained how the intervention would work including the stewarding, signage, and communication with parents and road users.

#### Control schools

School census data for primary schools in Bradford [23] was used to identify and match control schools based on (i) ethnicity, (ii) proportion of free school meals, and (iii) school size (Table 1). Ethnicity and free school meals (a proxy for deprivation) were chosen as they are associated with school travel choices [7]. In the UK, free school meals are available to pupils in receipt of, or whose parents are in receipt of, one or more types of government benefit/financial support and is not affected by school-level factors. For each intervention school, six to eight control schools were identified. Schools were subsequently prioritised based on their RAG rating (green and amber preferred to red). Due to the limited time available given the nature of the evaluation, amongst this remaining list we first contacted schools who were both amber or green and had taken part in previous research.

**Table 1:** Characteristics of Schools at Baseline based on the School Census (2022), with RAG rating

	Total Size (*n*)	White British children (%)	Free School Meals (%)	RAG Rating
School Street				
Intervention School 1	210–220	5–8%	26–29%	Green
Intervention School 2	415–425	87–90%	54–57%	Green
Intervention School 3	460–470	5–8%	28–31%	Green
Intervention School 4	685–695	11–14%	39–42%	Green
Control				
Control School 1	205–215	5–8%	29–32%	Amber
Control School 2	470–480	83–86%	44–47%	Green
Control School 3	425–435	7–10%	32–35%	Amber
Control School 4	615–625	4–7%	29–32%	Amber

School Street (intervention) and control schools with the same number are matched. Ranges are provided to prevent de-identification of schools

#### C-HaPIE questionnaire

Students completed the questionnaire online in a classroom during class time. Researchers attended each school to facilitate survey delivery; one school declined this offer due to scheduling difficulties. All questionnaires were administered using the REDCap electronic data capture tools hosted at University College London. The Child–Health and Place Intervention Evaluation (C-HaPIE) tool was developed for the research project based on Healthy Streets indicators [8, 24]. The C-HaPIE tool (Appendix 1) asked children about their perceptions of the built environment spanning the entire journey to school, including home street, journey and the road outside the school, as well as questions about travel mode to school and their wellbeing and health [25].

#### Additional School Street questions

Children in intervention schools were asked to rate the School Street intervention using a Likert scale. Children were first provided a written description of the School Street intervention with a photograph for illustration; the question “How much do you like the School Street project?” was then asked with potential responses “don’t know”, “not at all”, “a little”, or “a lot”. To discover whether other unanticipated outcomes emerged from the intervention and to provide context, at Time 1 and Time 2 children were asked two questions with free-text responses: (1) “What do you like about the School Street project?” and (2) “What do you not like about the School Street project?”.

#### Manual traffic counts

For one morning and afternoon and only during the restriction time, the number of motorised vehicles entering and leaving the restricted section of the school road was manually counted by a trained member of the Local Authority Highways team. Traffic was counted at baseline (May–June 2023) and again in July 2023 (Intervention Schools 1 and 2) and September 2023 (Intervention School 3), just after the schemes launched. Intervention School 3 had additional counts in June 2024.

### Outcome variables

#### Active travel

Active travel was assessed with three questions: (a) “How did you come to school today?” (car, walk, bike, bus, taxi, other; dichotomised as active (walk or cycle) or non-active travel mode (car, bus, taxi, other); (b) “Do you walk or cycle to school three or more days a week?” (yes, no; with ‘yes’ classified as “frequent active traveller”); (c) In an average school week, how many trips do you walk or cycle to school (range 0–10 visits)? Question (c) was calculated from four separate questions: in an average school week, how many days do you walk to school/from school; in an average school week, how many days do you cycle to school/from school (Appendix 2)?

#### Satisfaction with the school road

Satisfaction with the school road was assessed using a Likert scale, and the question “Overall, how much do you like the road outside your school?” was asked with potential answers: “very much”, “a little”, “not at all”. We grouped “a little” and “not at all” (Appendix 2).

#### Perceptions of road safety

Children’s perception of safety from traffic was assessed using a Likert scale, and the question “Do you feel safe crossing the road outside the school” was asked with potential answers: “not very safe”, “safe”, “very safe”. We grouped “safe” and “not very safe” (Appendix 2).

#### Control variables

Control variables were selected based on their associations with travel choices in prior research [7]. Children were asked about their gender (boy, girl, other/prefer not to say), ethnicity (Bangladeshi, black Caribbean, Pakistani, Chinese, white, mixed, Indian Asian, black African, other, prefer not to say), whether their family had a car (yes, no), and their school year (as a proxy for age). For gender, those who selected other/prefer not to say were excluded from the analysis due to low numbers (*n* = 23). Ethnicity was grouped as white (white), Asian (Bangladeshi, Pakistani, Chinese, Indian Asian) and all other ethnicities (mixed, black Caribbean, black African, other, prefer not to say) due to low numbers in some groups. The proportion of children reported to be receiving free school meals in the school census in 2022 and 2023 was included as a school-level variable as a proxy measure for deprivation. Given the UK’s climate variability, weather conditions on the morning of the survey (rain: yes/no) were included as a school-level variable for each school, for active travel on the day of the survey (Appendix 3).

### Analysis

#### Descriptive and analytical statistics

We summarised survey data (counts or median and interquartile range). Percentage difference was calculated for before and after traffic counts. A χ^2^ test was used to compare categorical data (active travel), and a Kruskal Wallis test was used to compare ordinal response data (satisfaction with the school road, perceptions of safety) between control and School Street schools at each timepoint. A Mann–Whitney U test was used to compare the number of trips between control and School Street schools across time points. A χ^2^ test was used to compare responses for the question “how much do you like the School Street?” across timepoints (Time 1 (October 2023) and Time 2 (May 2024)). This was performed on the total sample of intervention schools and at each site. A *p*-value < 0.05 was considered statistically significant. Statistical analysis was conducted using Stata v17.0 (Statacorp).

#### Difference-in-differences

We used ordinary least squares regression models with an interaction term between the time period (baseline (May 2023), Time 1 (October 2023), Time 2 (May 2024)), with baseline as the reference value, and an indicator variable for the intervention/control status of the school. We did a difference-in-difference analysis for each intervention school separately, with each intervention school compared to all four control schools. We repeated this procedure for the three outcomes (active travel, perceptions of the school road, and feelings of safety). We adjusted models for school year (as a proxy for age), gender, ethnicity, presence of a family car, and proportion of children receiving free school meals, to control potential confounding if these variables change between time points to different degrees in intervention and control schools. Travel mode on the day of the survey was additionally adjusted for weather conditions that day. We pooled the results from the three intervention schools using random effects meta-analysis to estimate an average effect of the intervention across the three schools.

#### Content analysis

Free text questions were analysed using content analysis [26] to determine the number of changes School Streets brought about and how strongly children felt about the changes. Data from Time 1 responses (*N* = 280) was used to inform the development of a coding framework [27]. This was applied deductively to qualitative data collected at Time 2. Data was coded by one author (SAH).

## Results

**Fig. 1:**
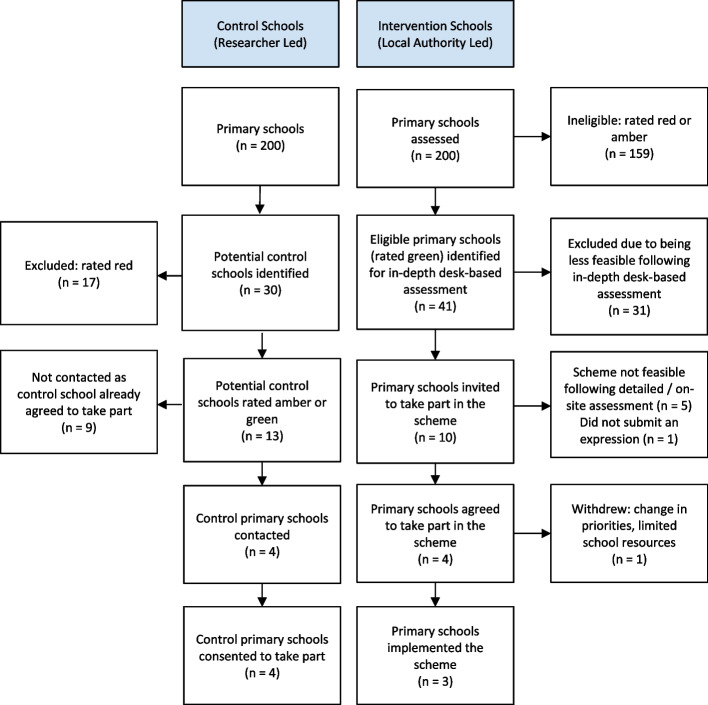
Diagram showing the selection and recruitment process of primary schools that participated in the study

The Local Authority RAG-rated 200 primary schools for feasibility of the School Streets scheme. Forty-one were identified as ‘green’ or schools where the scheme was likely feasible (Fig. 1). Following more in-depth desk-based assessment of feasibility, 10 schools were invited to submit an expression of interest. Amongst these 10 schools, council officers and school senior leaders subsequently concluded that the scheme was not feasible at five schools, one school did not submit an expression interest in the scheme. Four schools agreed to participate in the scheme. One intervention school withdrew after receiving the traffic order but before initiating the scheme, leaving three intervention and four control schools. Researchers identified four matched schools and invited them to participate as controls, and all four agreed.

### Characteristics

Across seven schools, 942 children had complete data for all control variables at Baseline (control *n* = 560, School Streets *n* = 382), 629 at Time 1 (control *n* = 372, School Streets *n* = 257), and 608 at Time 2 (control *n* = 382, School Streets *n* = 226); however, there were small differences in the number of children who responded to each outcome variable (Table 2). There was an even distribution of children across age groups and genders within control and School Street schools at all time points (*p* > 0.05; Table 2). There were differences in self-reported ethnicity between control and School Street schools at all time points (e.g., baseline: control schools 54% Asian, 21% white, 25% all other ethnic groups; School Street schools 36% Asian, 27% white, 37% all other ethnic groups; χ^2^ *p* < 0.001). Compared to control schools, reported family car ownership was higher in School Street schools at Time 1 (85 vs 79%, *p* = 0.049) and Time 2 (79 vs. 87%, *p* = 0.026).

**Table 2:** Characteristics of participants at all timepoints

	Baseline (May 2023)					Time 1 (October 2023)					Time 2 (May 2024)				
	Control		School Street			Control		School Street			Control		School Street		
	*n*	(%)	*n*	(%)	*p*	*n*	(%)	*n*	(%)	*p*	*n*	(%)	*n*	(%)	*p*
School Year															
Year 4 – age 8/9	177	(31.6)	137	(35.9)											
Year 5 – age 9/10	183	(32.7)	125	(32.7)		183	(49.2)	132	(51.4)		198	51.8	116	(51.3)	
Year 6 – age 10/11	200	(35.7)	120	(31.4)	0.291	189	(50.8)	125	(48.6)	0.593	184	(48.2)	110	(48.7)	0.904
Gender															
Girls	273	(48.8)	205	(53.7)		185	(49.7)	132	(51.4)		183	(47.9)	107	(47.4)	
Boys	287	51.3	177	46.3	0.138	187	50.3	125	48.6	0.688	199	52.1	119	52.7	0.742
Ethnicity															
Asian	300	(53.6)	137	(35.9)		178	(47.9)	89	(34.6)		197	(51.6)	93	(41.2)	
White	119	(21.3)	102	(26.7)		75	(20.2)	68	(26.5)		74	(19.4)	56	(24.8)	
Other	141	(25.2)	143	(37.4)	0.000	119	(32)	100	(38.9)	0.004	111	(29.1)	77	(34.1)	0.042
Family Car															
Yes	449	(80.2)	317	(83)		294	(79)	219	(85.2)		304	(79.6)	196	(86.7)	
No	111	(19.8)	65	(17)	0.278	78	(21)	38	(14.8)	0.049	78	(20.4)	30	(13.3)	0.026
Outcome Variables															
Active Travel (on day)															
Yes	292	(53.1)	258	(67.2)		196	(53.3)	156	(61.4)		217	(57.3)	130	(58.6)	
No	258	(46.9)	124	(32.8)	0.000	172	(46.7)	98	(38.6)	0.044	162	(42.7)	92	(41.4)	0.755
Active Travel (3 + days)															
Yes	281	(52.8)	240	(64.3)		176	(50.4)	150	(61.7)		186	(50.7)	131	(58.7)	
No	251	(47.2)	133	(35.7)	0.001	173	(49.6)	93	(38.3)	0.007	181	(49.3)	92	(41.3)	0.057
Active Travel (# of trips); median (IQR)*	8	(9)	10	(6)	0.001	8	(8)	10	(6)	0.074	8	(7)	10	(6)	0.210
Like road outside school															
Very much	131	(24.3)	81	(21.5)		106	(29.6)	77	(30.6)		83	(22.3)	51	(23.0)	
A little	348	(64.4)	238	(63.1)		208	(58.1)	149	(59.1)		235	(63.2)	146	(65.8)	
Not at all	61	(11.3)	58	(13.0)	0.092	44	(12.3)	26	(10.3)	0.535	54	(14.5)	25	(11.3)	0.458
Feel safe crossing															
Very Safe	239	(43.6)	156	(41.2)		170	(46.8)	125	(49)		138	(36.7)	100	(45.7)	
Safe	253	(46.2)	191	(50.4)		165	(45.5)	120	(47.1)		210	(55.9)	107	(48.9)	
Not very	56	(10.2)	32	(8.4)	0.730	28	(7.7)	10	(3.9)	0.363	28	(7.5)	12	(5.5)	0.028

^*^Active Travel (number of trips)—Baseline (*n* = 842), Time 1 (*n* = 527), Time 2 (*n* = 543)

In School Street schools, a higher percentage of children reported travelling to school actively on the day of the survey at baseline (67% vs. 53%; χ^2^ *p* < 0.001) and Time 1 (61% vs. 53%; χ^2^ *p* = 0.044) than in control schools. In School Street schools, a higher percentage of children identified themselves as frequent active travellers (≥ 3 days/week) at baseline (64% vs. 53%; χ^2^ *p* = 0.001) and Time 1 (62% vs. 50%; χ^2^ *p* = 0.007) than in control schools. Compared with children in control schools, children in School Street schools reported a higher number of active trips to and from school at baseline (median ± IQR 10 ± 6 vs. 8 ± 9; *p* < 0.001). There were no statistically significant differences in any measure of self-reported active travel between school groups at Time 2.

Children’s perceptions of liking the road outside their school was similar across School Street and control schools at all time points. At Time 2, differences between children’s perception of safety crossing the road outside the school were observed between control (very safe 37%, safe 56%, not very safe 8%) and School Street (very safe 46%, safe 49%, not very safe 6%; *p* = 0.028) schools.

### Manual traffic counts

Intervention Schools 1 and 2 had a 60–90% reduction in traffic volumes at Time 1; Intervention School 3 had a 94–95% reduction in September 2023 and a 23–73% reduction in traffic volume in June 2024.

### Active travel

**Fig. 2:**
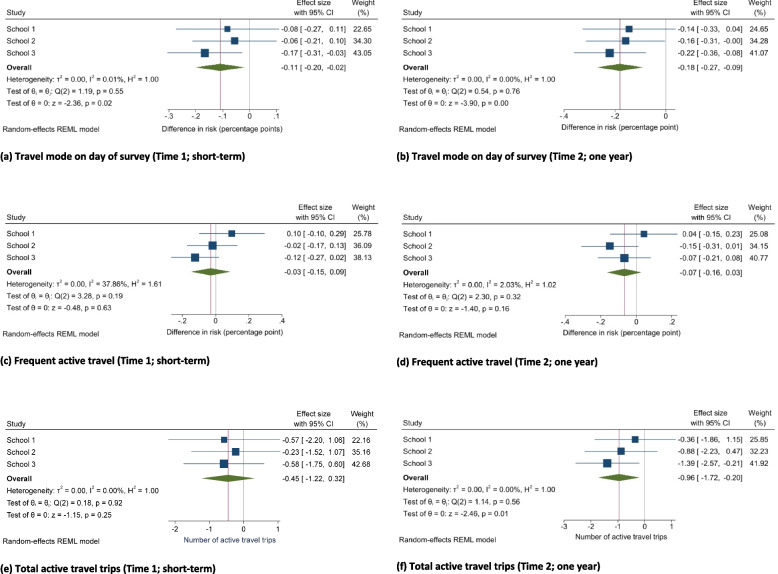
Difference-in-differences effect estimates and confidence intervals for changes in self-reported active travel “How did you come to school today?” (walk, bike vs. car, bus, taxi, other; dichotomised as active or non-active travel) at Time 1 (**a**) and Time 2 (**b**); “Do you walk or cycle to school 3 or more days a week?” (yes, no; “frequent active travel”) at Time 1 (**c**) and Time 2 (**d**); “In an average school week how many trips do you walk or cycle to school” (range 0–10 trips) at Time 1 (**e**) and Time 2 (**f**). All models were controlled for school year, gender, ethnicity, presence of a family car, and proportion of children within the school eligible for free school meals. Travel mode on the day of the survey was additionally adjusted for weather conditions that day (rain/storm or dry). Fewer children had complete data for total active travel trips: Baseline (*n* = 842), Time 1 (*n* = 527), Time 2 (*n* = 543)

Compared to the change in active travel on the day of the survey in control schools, active travel decreased in School Street schools at Time 1 (B −0.11, 95% CI −0.20, −0.02; *p* = 0.02) and Time 2 (B −0.18, 95% CI −0.27, −0.09; *p* < 0.001) (Fig. 2a,b). We did not find evidence of a change in the number of children identifying themselves as frequent active travellers between the control and School Street schools at Time 1 (B −0.03, 95% CI −0.15, 0.09; *p* = 0.63) and Time 2 (B −0.07, 95% CI −0.16, 0.03; *p* = 0.16) (Fig. 2c,d). Compared to the change in the total number of active travel trips in control schools, the absolute number of reported active travel trips significantly decreased in the School Street schools at Time 2 (B −0.96, 95% CI −1.72, −0.20; *p* = 0.01) but not Time 1 (B −0.45, 95% CI −1.22, 0.32; *p* = 0.25) (Fig. 2e,f).

### Satisfaction with the school road

**Fig. 3:**
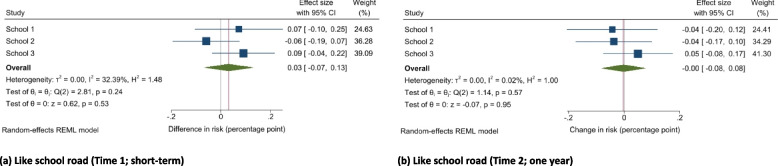
Difference-in-differences effect estimates and confidence intervals for changes in reported satisfaction with the school road Do you like the road outside your school (not at all/a little or a lot) at Time 1 (**a**) and Time 2 (**b**). All models were controlled for school year, gender, ethnicity, presence of a family car, and proportion of children within the school eligible for free school meals

Compared to the change in children liking the road outside their school in control schools, we did not find evidence of a difference at Time 1 (B 0.03, 95% CI −0.07, 0.13; *p* = 0.53) or Time 2 (B 0.00, 95% CI −0.08, 0.08; *p* = 0.95) (Fig. 3a,b).

### Perceptions of road safety

**Fig. 4:**
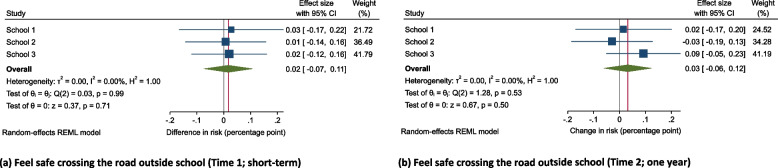
Difference-in-differences effect estimates and confidence intervals for changes in self-reported perceptions of safety Do you feel safe crossing the roads outside the school (not very safe/safe or very safe) at Time 1 (**a**) and Time 2 (**b**). All models were controlled for school year, gender, ethnicity, presence of a family car, and proportion of children within the school eligible for free school meals

Compared to the change in children’s perception of feeling safe crossing the road outside their school in control schools, we did not find evidence of a difference in children’s perceptions at Time 1 (B 0.02, 95% CI −0.07, 0.11; *p* = 0.71) or Time 2 (B 0.03, 95% CI −0.06, 0.12; *p* = 0.50; Fig. 4a,b) in School Street schools.

### Children’s perceptions of School Streets

Across the three intervention schools, children reported how much they liked the School Street intervention in October 2023 (T1; *n* = 263) and May 2024 (T2; *n* = 265) (Table 3). We did not find evidence of a difference in perceptions of School Streets between October and May (*p* = 0.53). When examined by school, perceptions of the School Street intervention only varied in one school (Intervention School 2) between time points (*p* = 0.04). It was hypothesised that the change observed in Intervention School 2 related to modifications in its implementation of the School Streets scheme, whereby the road closures were only enforced by signage and not by volunteers (school staff) after the October survey. This hypothesis, gained from anecdotal evidence from the school as well as the data shown in Table 3 was used to refine the coding framework in the content analysis to analyse responses to the free text questions collected in May 2024.

**Table 3:** Children’s Perceptions of School Streets (“Do you like School Streets?”)

Response	Oct 2023 (T1) *n* (%) (*n* = 263)	May 2024 (T2) *n* (%) (*n* = 265)	Change (percentage points)
A lot	60 (23)	45 (17)	- 6
A little	71 (37)	114 (43)	+ 6
Not at all	47 (18)	45 (17)	- 1
I don’t know	42 (16)	48 (18)	+ 2

In May 2024, 229 pupils completed the free text question “What do you like about School Streets?” and 227 completed the free text question “What do you not like about School Streets?”. Children liked that School Streets increased (i) feelings of solidarity to protect children from road safety issues and (ii) the perception of safety by reducing the number of vehicles outside schools (Table 4). Children perceived barriers to car travel associated with School Streets as they did not like that the scheme was inconvenient for drivers and that traffic congestion remained outside the School Street zone. This indicates that some children did not perceive a widespread change in travel mode from using private vehicles and that School Streets did not challenge the belief that streets are just for cars (Table 4).

**Table 4:** Key themes identified from free text analysis of children’s survey responses

Theme	Description	Example Verbatim Quotes
Theme 1: School Streets increased feelings of solidarity to protect children from road safety issues		
School leaders are enacting a collective responsibility for children’s safety	The school (or staff) are present at the School Street entrance to keep pupils safe	“That the School is trying to keep us safe and that they care about us…”—Year 6 Pupil
Children value the School Street because they recognise that others might need protection from road traffic	The School Street protects vulnerable road users such as younger children, people with disabilities, grandparents and children who walk to school independently	“I like that there closing the streets down making it safe for young children to cross the road.”—Year 6 Pupil
Theme 2: School Streets improved the perception of safety by reducing the number of vehicles outside schools		
A reduction in the number of motor vehicles	Improved perceptions of safety for pedestrians	“It keeps cars away”—Year 5 Pupil
There are fewer opportunities for pedestrian injury	A reduction in vehicles also reduces the chance of a child pedestrian injury	“It reduces the amount of danger that can occur by children getting run over by cars”—Year 5 Pupil
Improved pedestrian confidence when crossing the road and walking	Fewer vehicles and less threat of injury improve children’s confidence in the area outside their School	“Yay no more disturbing cars in my way!!!!!!I can finally cross the road and be safe!”—Year 6 Pupil
A more peaceful atmosphere	The School Street has made being a pedestrian outside the school less stressful	“that the road is peacefull to walk across to school.”—Year 6 Pupil
Theme 3: Children perceived barriers to car travel associated with School Streets		
School Streets are inconvenient for those who drive to school	The School Street did not change some pupils’ travel mode	“If someone wants to come to school in a car (if they live far away) there isn't much parking space so they might have to park far away or if there late for work they can't get past”—Year 5 Pupil
School Streets create traffic congestion beyond the restricted zone indicating that others are not changing their travel mode	Children attributed growing traffic congestion to the School Street	“It causes traffic on the part thats not blocked.”—Year 6 Pupil
School Streets block the roads off to most motor vehicles	Some children maintained a car-centric view of the space outside the school	“because it takes up space from when I need to go through that exact road, its blocked so it is very annoying”—Year 5 Pupil

The most significant theme that emerged related to the effect School Streets had on increasing solidarity between children and adults. The presence of school leaders at the School Street entrances demonstrated how they took collective responsibility for children’s road safety. This key change led to an improved perception of safety as their presence ensured a reduction in motor vehicles, which meant fewer opportunities for pedestrian injury. This improved pedestrian confidence when crossing the road and walking, and children felt that the road outside their school was more peaceful.

However, the most prominent theme arising from the question which asked children what they did not like about School Streets was that the scheme presented challenges for vehicle drivers. This theme arose directly from pupils who stated that School Streets are inconvenient for car drivers, that they block the roads off to motor vehicles and that traffic congestion remained outside the School Street sites.

## Discussion

This study aimed to determine whether School Streets led to increased active travel, perceptions of safety from traffic, and satisfaction with the school road amongst children. At baseline, active travel in intervention schools was higher than regional and national estimates and when compared with control schools. The number of children that reported actively travelling on the day of the survey was reduced in School Street schools compared to those in control schools at approximately one month and one year after implementation. The average number of active trips completed reduced by 0.96 one year after baseline compared with control schools; however, the number of children identifying as frequent active travellers did not differ between school groups. Most children were satisfied with the street outside their school and felt safe crossing the road outside their school. We found no evidence that the School Streets intervention changed these outcomes.

### Active travel

Improving the uptake of active travel is one of the main reasons local authorities introduce School Street schemes [12, 14]. We did not find evidence of a change in the number of children identifying themselves as frequent active travellers, however we have counterintuitively shown that self-reported active travel on the day and the number of self-reported active travel trips were reduced in these schools, contrasting with expectations and suggestions from before-and-after evaluations [14, 16]. One before-and-after evaluation in London found that School Streets did not impact how children or parents travel to school; however, this was during the COVID-19 pandemic [28]. The reasons for our findings are unclear but may relate to the selection process for intervention schools, which may lead to potentially differing reasons for implementing the scheme between the Local Authority and schools. Moreover, the selection was determined based on the feasibility criteria of the School Street scheme. This meant that the surrounding streets of the intervention schools already had characteristics favourable to promoting active travel to school, such as cul-de-sacs or low-traffic routes [29]. In line with this, we found that School Street intervention schools had higher levels of self-reported frequent active travel at baseline (64%) compared with control schools (53%) (which were both amber and green rated) and in comparison with both national (51%) and regional (44%) averages [30]. Whilst cautious comparison is required with national and regional estimates due to differences in the phrasing of questions and the age groups used, self-reported frequent active travel was greater than both younger (5–10 years) and older (11–16 years) children in the National Travel Survey [30]. This finding of an above-average baseline in active travel has also been seen in another School Street evaluation [28], suggesting that recruitment (e.g. self-selection of intervention schools, eligibility criteria) may not target schools with the highest need or scope for active travel interventions. Therefore, at such high levels of active travel there may have been limited opportunity to improve the uptake of active travel as a result of the intervention. Other factors which may have affected active travel include possible displacement of traffic congestion to surrounding areas making active travel less attractive, parental concerns leading to changes in school-run behaviours, a potential change in transport patterns due to post-COVID-19 normalisation.

### Safety

School Streets did not affect children’s perceptions of safety (crossing the road outside their school). This contrasts with evidence in the literature; however, these studies focused on adults (parents, teachers and residents) who reported that the streets were safer [16–18]. Although road safety and traffic congestion were motivators for trialling School Streets in previous Bradford schools [27], most children reported feeling safe at all time points, suggesting that perhaps, unlike adults [7], they did not perceive this problem. This may be because most children were in familiar environments and accompanied by an adult who may have attenuated their concerns or shielded them from danger [31], particularly when crossing roads to which this question pertained. However, highlighting the nuance and multi-dimensionality in interpreting road safety, in their free text responses children reflected that they felt adults were working to protect them from road safety issues. The children related these efforts to more tangible results of the intervention, including fewer cars on the street, a reduced risk of injury, a more peaceful environment, and greater confidence when walking. Whilst evidence in this area is limited, others have similarly found that road closures for play (Play Streets) can lead to a greater sense of connectedness among children and parents and improved feelings of safety amongst parents [32].

### Satisfaction

School Streets did not affect children’s satisfaction with their school road. Although children broadly approved of the School Streets intervention, there was no change in satisfaction with the road outside the school. We hypothesise that this may relate to how children perceive School Streets, not as the physical road but as the adults working together and a sense of solidarity to protect children from road safety issues. Moreover, in our previous work [24] and consistent with the Healthy Streets indicators [8], we identified that having things to see and do on the way to school was important to children’s wellbeing. As the scheme was introduced without complementary changes to the built environment, it is perhaps unsurprising that children’s perceptions of the physical street did not change.

### Intervention

Schools eligible for a School Street scheme were those where it was feasible to restrict motorised traffic on the street rather than schools that had the greatest need, for example, those with high levels of air pollution or low active travel uptake due to proximity to major roads. Difficulties implementing School Streets due to lack of enforcement and motorist compliance are also well documented [14, 16]. Whilst monitoring the implementation of School Streets was not a component of this study, anecdotal reports from the Local Authority and school staff identified that volunteers had stopped stewarding the intervention in one school. Although similar issues were not identified amongst the other schools, it is possible that variations in implementation may have occurred. Single component interventions such as traffic restrictions imposed on short lengths of road without complementary interventions (e.g. walking school bus, park and stride scheme, cycle loan scheme, improved infrastructure) do little to challenge the root causes of car travel (e.g. trip chains [7]) and tend to be less likely to succeed than a multi-level approach [33]. This was also observed by the children in this study, who felt that the intervention did not change travel mode for many, as many still viewed the street outside their school as being for cars and felt that the intervention led to congestion and inconvenience for car drivers. Further work is required to clarify the theory of change underlying School Streets.

### Strengths and limitations

There are several strengths to this study. To the best of our knowledge, this is the first study to use a controlled study design to estimate the effects of School Streets on active travel, safety and satisfaction with the school road. A year-long design was adopted to account for seasonal variations. The study was designed and delivered in close collaboration with the Local Authority, resulting in a greater understanding of the theory of change underpinning their implementation of the School Streets intervention and subsequent outcomes we sought to measure, as well as a high level of buy-in amongst schools for the evaluation. A further strength of this study is our focus on children and amplifying the children’s voices, which have so far been under-represented in this area. The limitations of this study must also be acknowledged. The study has five key limitations: (1) a small number of schools took part whose demographics were not reflective of Bradford’s average population, which meant our estimates had limited precision and generalisability; (2) we have not collected data on implementation; (3) there may be measurement error and it is unknown how accurately children can recall ‘usual’ travel habits (note we do not think this is likely to be differential between intervention and control schools, therefore measurement error is most likely to bias effect estimates towards the null); (4) the intervention schools had a high level of active travel at baseline, which was not reflective of regional or national patterns; (5) we were unable to explore the parallel trends assumption for the difference in difference analysis, as we did not have longer time-series of our outcome measures. As discussed above, differing background trends might bias or explain some of our results, for example if intervention schools had a longer-term trend of reducing active travel while control schools did not, this may explain the apparent reduction in active travel associated with the intervention. Although we did not have evidence for the plausibility of the parallel trends assumption, we do not have any strong reason to think that trends in active travel would differ widely between the schools included in this study.

## Conclusion

Using a controlled study design, we saw no evidence that the implementation of a School Streets intervention affected children’s perceptions of feeling safe or satisfied with their school road and in fact observed some decreases in active travel. This reduction may be due to chance; biases such as differing trends in active travel between intervention and control schools, or differing changes in measurement error; or residual confounding in which unmeasured pupil characteristics changed differentially between intervention and control schools. Qualitative data found that children felt safer due to less traffic on the streets, highlighting a nuance in perceptions of road safety amongst children. A novel finding of this study is the sense of solidarity and community cohesion that School Streets elicits. Future research should investigate the effects of School Streets interventions in roads with environments unconducive to active travel (i.e., proximity to busy roads), monitor the implementation of the schemes and undertake a more detailed assessment of travel behaviour (e.g. travel diaries considering multiple travel modes per trip and reasons for travel mode). Moreover, researchers should seek to better understand how School Streets affects children (e.g. safety, solidarity, community cohesion). This information is important for decision-makers to understand the theory of change underlying School Streets and whether it is a cost-effective intervention for the outcome they seek to affect. In practice, schools should be made aware of how the presence of volunteers is positively perceived by children and consider whether complementing the intervention with other measures (e.g. walking and cycling infrastructure, things to see and do) is feasible and whether it promotes active travel and satisfaction with their school road and safety.

## Supplementary Information

Supplementary Material 1.

Supplementary Material 2.

Supplementary Material 3.

## Data Availability

The datasets used and/or analysed during the current study are available from the corresponding author on reasonable request.
