# Supplementary material for: The effect of a School Street intervention on children’s active travel, satisfaction with their street, and perception of road safety: a natural experimental evaluation
Source: BMC Public Health. 2025 Jul 2;25:2207. doi: 10.1186/s12889-025-23236-8 (PMC12219895; doi:10.1186/s12889-025-23236-8)
Supplement: Supplementary file 1 — Supplementary Material 1. [file 12889_2025_23236_MOESM1_ESM.pdf]

YOUNG PEOPLES TRAVEL & ACTIVITY

YOUR CONSENT

By completing this survey you are agreeing to take part in this study and for your data to be stored at UCL.

These data will be used to find out if changes to the streets are related to children's health and wellbeing. The data may be reported in an academic journal or at conferences. You will not be identified in any such report. You can withdraw from the study by not submitting the questionnaire. Data from unfinished questionnaires will not be included in analyses.

Do you wish to continue to the survey?

Yes ☐

No ☐

Please tick the box next to your answer

Please try to answer all the questions. There are no right or wrong answers. We want to know what you think. Your answers will not be shown to anyone that you know (including mums and dads).

If you do not want to answer a question you can miss it out.

If you need any help, you can ask your teacher or the person who gave you this.

Thank you!

ABOUT YOU

1. Which school year are you in? ***(Please tick ONE box)***

Year 5 ☐

Year 6 ☐

2. What is the name of your School?

WRITE HERE:

3. Are you ***(Please tick ONE box)***

a girl ☐

a boy ☐

4. How would you describe yourself: ***(Please tick ONE box)***

White ☐

Black African ☐

Black Caribbean ☐

Indian Asian ☐

Pakistani ☐

Bangladeshi ☐

Chinese ☐

Mixed ☐

Other ☐

WHERE YOU LIVE

5. What is the name of the street you live in?

WRITE HERE:

6. Would you say you are happy where you live? ***(Please tick ONE box)***

- Very happy ☐
- Quite happy ☐
- Not very happy ☐

7. How long have you been living in this area? ***(Please tick ONE box)***

- Less than a year ☐
- 1-3 years ☐
- More than 3 years ☐

8. How safe do you feel crossing the road where you live? ***(Please tick ONE box)***

- Very safe ☐
- Quite safe ☐
- Not very safe ☐

9. How noisy is the traffic on the road where you live? ***(Please tick ONE box)***

- Very noisy ☐
- Quite noisy ☐
- Not very noisy ☐

10. Are there trees and plants on the road where you live? **(Please tick ONE box)**

Many ☐

Some ☐

Not many ☐

11. Are there nice things to see and do on the road where you live? **(Please tick ONE box)**

Many ☐

` Some ☐

Not many ☐

12. Does your home have a garden where you can play?

Yes ☐

No ☐

13. Does your family have a car?

Yes ☐

No ☐

14. Do you have access to a bicycle in your house?

Yes ☐

No ☐

ABOUT YOUR SCHOOL JOURNEYS

Your trip TO school this morning

15. How did you come to school today? ***(Please tick ONE box for the main way you travelled)***

Car 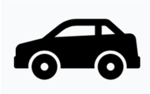 ☐

Walk 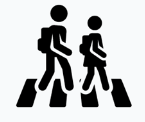 ☐

Bike 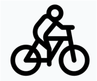 ☐

Bus 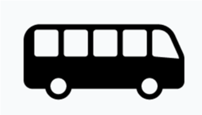 ☐

Taxi 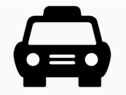 ☐

Other write in ☐

16. How many roads did you have to WALK across on your way to school? (e.g. 0, 1, 2.) **Write In number** ☐

17. **How long did it take you to get to school today?**

1 to 5 minutes ☐

6 to 10 minutes ☐

11 to 15 minutes ☐

16 to 20 minutes ☐

21 to 30 minutes ☐

More than 31 minutes ☐

18. Who came with you on your trip to school this morning? ***(Please tick a box for everyone who came with you)***

Alone ☐

Parent/other relative ☐

Other adult ☐

Brother or sister ☐

Friends ☐

19. How will you travel back home from school today? ***(Please tick ONE box for the main way you will travel)***

Car 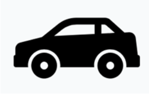 ☐

Walk 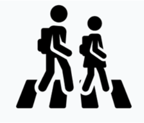 ☐

Bike 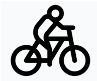 ☐

Bus 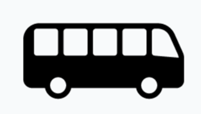 ☐

Taxi 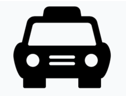 ☐

Other write in ☐

20. Did you feel happy and relaxed on your trip to school today?

Very happy and relaxed 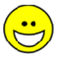 ☐

Quite happy and relaxed ☐

Not very happy and relaxed at all ☐

21. How safe from **traffic** did you feel on your trip to school today?

Very safe ☐

Quite Safe ☐

Not very safe at all ☐

22. How safe from **crime** did you feel on your trip to school today?

Very safe ☐

Quite Safe ☐

Not very safe at all ☐

23. Are the pavements on your trip to school in good condition?

Very good ☐

Quite good ☐

Not very good at all ☐

24. Overall, how much do you like your journey to school? (Please tick ONE box)

Very much ☐

A little ☐

Not at all ☐

25. Do you walk or cycle to school 3 or more days a week

Yes ☐

No ☐

In an average school week, on how many days do you **walk to get to school?** (e.g. 0,1,2,3,4,5) **Write In number**	☐
In an average school week, on how many days do you **cycle** to get to school? (e.g. 0,1,2,3,4,5) **Write In number**	☐
In an average school week, on how many days do you **walk back home from school?** (e.g. 0,1,2,3,4,5) **Write In number**	☐
In an average school week, on how many days do you **cycle back home from school?** (e.g. 0,1,2,3,4,5) **Write In number**	☐
**If currently you do not walk or cycle 3 or more days to school, would you like to?**	
Yes	☐
No	☐

What would make it easier for you to walk or cycle to school? **Write In**	

OUTSIDE YOUR SCHOOL

26. How safe do you feel crossing the road outside your school? ***(Please tick ONE box)***

Very safe ☐

Quite Safe ☐

Not very safe at all ☐

27. How noisy is the traffic on the road outside your school? ***(Please tick ONE box)***

Very noisy ☐

Quite noisy ☐

Not very noisy at all ☐

28. Are there enough places to play near your school?

Enough ☐

Some ☐

Not enough ☐

29. Are there trees and plants on the road outside your school? ***(Please tick ONE box)***

Many ☐

Some ☐

Not many ☐

30. Are there nice things to see and do around your school? *(Please tick ONE box)*

Many ☐

Some ☐

Not many ☐

What sort of things would make the area around your school nicer to be in?
Write In

31. How clean do you think the air is on the road outside your school?

Very clean ☐

Quite clean ☐

Not very clean at all ☐

32. Overall, how much do you like the road outside your school

Very much ☐

A little ☐

Not at all ☐

ACTIVITIES

33. Last week, did you play or hangout in a park near you?

Yes ☐

No ☐

34. Last week, did you play or hangout in the streets near you?

Yes ☐

No ☐

HOW YOU FEEL

Please try to answer all of these questions. Remember there are no right or wrong answers. We want to know what you think. Your answers will not be shown to anyone that you know (including mums and dads).
If you do not want to answer a question you can miss it out.

35. How often do you feel happy?

All of the time ☐

Some of the time ☐

Never ☐

Prefer not to say ☐

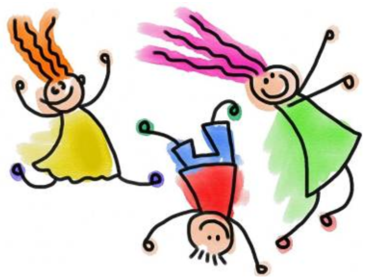

36. How often do you feel sad?

All of the time ☐

Some of the time ☐

Never ☐

Prefer not to say ☐

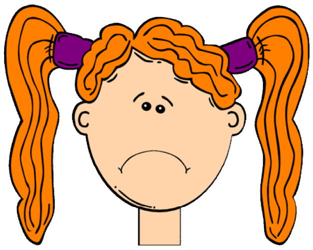

37. How often are you ill or unwell?

All of the time ☐

Some of the time ☐

Never ☐

Prefer not to say ☐

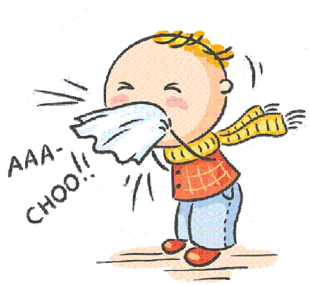

38. How often do you feel healthy?

All of the time ☐

Some of the time ☐

Never ☐

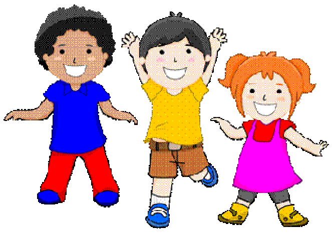

Prefer not to say ☐

FRIENDS

39. How many friends do you have?

Lots ☐

Some ☐

Not many ☐

Prefer not to say ☐

That's the end of the questions thank you for your help!
