# Supplementary material for: The effect of a School Street intervention on children’s active travel, satisfaction with their street, and perception of road safety: a natural experimental evaluation
Source: BMC Public Health. 2025 Jul 2;25:2207. doi: 10.1186/s12889-025-23236-8 (PMC12219895; doi:10.1186/s12889-025-23236-8)
Supplement: Supplementary file 2 — Supplementary Material 2. [file 12889_2025_23236_MOESM2_ESM.pdf]

Appendix 2: Data cleaning and Dichotomisation

Data Cleaning - Total Active Travel Trips Variable

To estimate the number of trips children completed in an average week (in an average school week how many trips do you walk or cycle to school (range 0 – 10 trips)), the responses of four questions were summed:

1. In an average school week, on how many days do you walk to school? [Open text]
2. In an average school week, on how many days do you walk to from school? [Open text]
3. In an average school week, on how many days do you cycle to school? [Open text]
4. In an average school week, on how many days do you cycle from school? [Open text]

The total active travel trips variable was marked missing if participants had missing responses for one or more of the four questions.

Due to an error on the online survey whereby an open text response rather than numerals was enabled, each of these questions were individually cleaned. The following process was adopted:

1. Numbers written as text (e.g. "five", "o", or "five days") and phrases (e.g. "every day", "none") were converted to numerals.
2. Numbers between 6-10 were changed to 5. This approach was taken due to feedback from the field researchers who observed that some children wrote 'seven' instead of 'five'; possibly due to associations with the word 'week' in the question.
3. Answers with a range (e.g. 2-3 days) were converted to the lowest number to avoid over-estimation.
4. Any responses that were unclear (e.g. '1 million', 'I don't know' or '1 2 3 4 5') or were greater than 10 were marked as missing.

Once the responses to all four questions were cleaned, they were added together to create the variable for total active travel trips. Most responses fell between 0-10 trips; however, children who reported that they completed more than 10 active travel trips had their responses reduced to the maximum of 10 trips (3-5%). The number and reasons for modifications to the children's responses is shown in **Table S1** below.

Table S1: Modifications made to the four variables used to calculate the total number of active travel trips

	**May 2023** (3988 responses)	**October 2023** (2660 responses)	**May 2024** (2616 responses)
Missing responses	134 (4%)	251 (10%)	168 (7%)
**Modifications**			
Total modified responses	339 (9%)	350 (14%)	184 (8%)
Reasons for modifications			
Phrase / text	183 (5%)	168 (6%)	105 (5%)
Number between 6-10*	36 (1%)	11 (0.4%)	17 (1%)
Number greater than 10#	25 (1%)	23 (1%)	5 (0.2%)
Range provided	54 (1%)	38 (2%)	28 (1%)
Unclear response#	41 (1%)	110 (4%)	29 (1%)
**Modification (generated variable)**	**May 2023** (n = 942)	**October 2023** (n = 629)	**May 2024** (n = 608)
Total > 10, reduced to 10	44 (5%)	27 (4%)	19 (3%)
**Missing (generated variable)**			
Missing	100 (11%)	102 (16%)	65 (10%)

* Changed to ‘5’; # marked as missing.

Dichotomisation

Table S2: Re-categorisation of outcome variables

	Question	Responses / Likert Scale	Re-categorisation
1	How did you come to school today?	Car, walk, bike, bus, taxi, other;	Active (i.e. walk, bike) or non-active travel mode (car, bus, taxi, other)
2	Overall, how much do you like the road outside your school	Very much, a little, not at all	Very much vs. a little or not at all
3	Overall, how much do you like your trip to school?	Very much, a little, not at all	Very much vs. a little or not at all
4	Do you feel safe on your journey to school	Very safe, safe, not very safe	Very safe vs. not very safe, safe
5	Do you feel safe crossing the roads outside the school	Very safe, safe, not very safe	Very safe vs. not very safe, safe
