# Supplementary material for: The effect of a School Street intervention on children’s active travel, satisfaction with their street, and perception of road safety: a natural experimental evaluation
Source: BMC Public Health. 2025 Jul 2;25:2207. doi: 10.1186/s12889-025-23236-8 (PMC12219895; doi:10.1186/s12889-025-23236-8)
Supplement: Supplementary file 3 — Supplementary Material 3. [file 12889_2025_23236_MOESM3_ESM.pdf]

Appendix 3: Weather

Table S3: Temperature and weather conditions (rain or dry) on the day of the survey at each school

	May 2023<br>(Baseline)	October 2023<br>(Time 1)	May 2024<br>(Time 2)
**School Street**			
Intervention School 1	8°C, dry	15°C, dry	12°C, dry
Intervention School 2	12°C, dry	10°C, dry	(Day 1) 11°C, dry<br>(Day 2) 11°C, dry
Intervention School 3	10°C, dry	10°C, rain	12°C, dry
**Control**			
Control School 1	11°C, dry	12°C, dry	11°C, dry
Control School 2	12°C, dry	10°C, dry	11°C, dry
Control School 3	12°C, dry	11°C, rain	12°C, dry
Control School 4	17°C, dry	(Day 1) 8°C, dry<br>(Day 2) 10°C, rain	12°C, rain

Temperature was recorded by the researchers at the start of data collection using BBC Weather; weather conditions were based on researcher observations.
